# Network meta-analysis of (individual patient) time to event data alongside (aggregate) count data

**DOI:** 10.1186/1471-2288-14-105

**Published:** 2014-09-10

**Authors:** Pedro Saramago, Ling-Hsiang Chuang, Marta O Soares

**Affiliations:** 1 Centre for Health Economics, University of York, Heslington, York YO10 5DD, UK; 2 Pharmerit International, Rotterdam, The Netherlands

**Keywords:** Evidence synthesis, Time to event data, Survival data, Individual patient data, Aggregate data, Meta-analysis, Mixed treatment comparisons, Network-meta-analysis, Treatment-effect modifiers

## Abstract

**Background:**

Network meta-analysis methods extend the standard pair-wise framework to allow simultaneous comparison of multiple interventions in a single statistical model. Despite published work on network meta-analysis mainly focussing on the synthesis of aggregate data, methods have been developed that allow the use of individual patient-level data specifically when outcomes are dichotomous or continuous. This paper focuses on the synthesis of individual patient-level and summary time to event data, motivated by a real data example looking at the effectiveness of high compression treatments on the healing of venous leg ulcers.

**Methods:**

This paper introduces a novel network meta-analysis modelling approach that allows individual patient-level (time to event with censoring) and summary-level data (event count for a given follow-up time) to be synthesised jointly by assuming an underlying, common, distribution of time to healing. Alternative model assumptions were tested within the motivating example. Model fit and adequacy measures were used to compare and select models.

**Results:**

Due to the availability of individual patient-level data in our example we were able to use a Weibull distribution to describe time to healing; otherwise, we would have been limited to specifying a uniparametric distribution. Absolute effectiveness estimates were more sensitive than relative effectiveness estimates to a range of alternative specifications for the model.

**Conclusions:**

The synthesis of time to event data considering individual patient-level data provides modelling flexibility, and can be particularly important when absolute effectiveness estimates, and not just relative effect estimates, are of interest.

## Background

Treatment decisions in medicine, whether at the patient- or policy level, should consider all relevant alternative health care technologies potentially capable of delivering the change being sought. Such informed decision making on the use of competing treatments requires evidence of relative effects from randomised controlled trials (RCTs) (which may be further used to inform estimates of cost-effectiveness). However, trying to synthesise evidence from several different pair-wise comparisons relevant to a decision problem can be challenging. A piecemeal approach can be avoided if all RCTs evaluating interventions relevant to the treatment decision are considered collectively, for example, with the use of network meta-analysis (NMA). NMA is a well-established statistical technique that extends standard pairwise meta-analysis framework to allow simultaneous comparison of multiple interventions in a single statistical model [1,2]. This approach then produces relative effect estimates (and associated descriptions of uncertainty) for all treatments connected by the network of evidence [3] – even where head-to-head trials for comparisons do not exist.

### NMA using individual patient data

Published work on NMA mainly focuses on the synthesis of aggregate data (AD) (sometimes called summary data, e.g. group means and standard errors available from study reports) [4,5]; however, methods have been developed that allow use of individual patient-level data (IPD) in NMA, specifically for binary and continuous data [6-8]. The appeal of including IPD in a NMA is that it is likely to reduce statistical heterogeneity across the network (and in this way help resolve possible inconsistencies); it may also allow subgroup effects to be estimated which in turn could guide more personalised treatment decisions [6]. The use of IPD, alone or in combination with AD, has been shown to improve inference in NMAs where the outcome of interest is binary (dichotomous) by aiding convergence, and by providing unbiased treatment–covariate interactions (that would otherwise be affected by ecological bias [9]). For continuous outcomes, IPD is likely to produce more precise estimates of treatment effects, even in the absence of treatment–covariate interactions [10].

### NMA using time to event related outcome data

When undertaking an NMA, aggregate time to event data presented as hazard ratios (HR), and some measure of uncertainty around these estimates, can be pooled directly using standard methods (analogous to pooling odds ratios or relative risks) [11]. However, other AD outputs such as median/mean time to event [12] and cumulative counts of patients having the outcome event in a period of time [13] have also been meta-analysed in a network. In modelling these data, an underlying time to event distribution can be specified which will then allow HRs to be generated from the original AD [5,14,15]. To date, evidence synthesis that includes IPD on time to event has been limited to pairwise analysis; additionally there has been little methodological exploration how outcomes in the form of IPD and AD might be considered together in the same NMA [13].

### Developing a NMA combining AD and IPD data to synthesise time to event related outcomes

This paper describes a modelling framework that combines AD and IPD in the synthesis of time to event related outcomes within an NMA. It extends the work of Saramago et al. [6] and Sutton et al. [16] on the synthesis of AD and IPD for binary data to consider time to event outcomes. And extends the work on NMAs for time to event AD outcomes by Soares et al. [15] and Woods et al. [14] (the general modelling framework is also described in Dias et al. [5]). Our work was motivated by an NMA on treatments for healing venous leg ulcers, for which we had data from multiple RCTs (more information provide in the next section). A proportion of the RCT data on ulcer healing was available in IPD format (time to healing and time to censoring), with the remaining data available as AD, i.e. count data (proportion of patients healed at end of follow-up which differs across studies). To maximally draw from available data we aimed to jointly synthesise the available AD and IPD, and, in this way, generating better estimates and providing fuller characterisations of uncertainty to best inform decisions on the use of the treatments of interest.

### Motivating example: high compression treatments for venous leg ulcers

The case study relates to compression systems aiming to deliver high compression (classed as ≥40 mmHg compression at the ankle) to promote venous leg ulcer healing. Available standardised systems are: two layer hosiery (HH), the four layer bandage (4LB), the short stretch bandage (SSB), the zinc paste bandage (ZINC), and the two layer bandage system (2LB). A detailed description is provided in Additional file 1 with further details of these systems presented elsewhere [17].

Effectiveness evidence from RCTs was obtained from the most recent update of the relevant Cochrane review available to us, and from a recent multicentre RCT which compared 4LB with HH. All available RCT evidence was assessed for inclusion in the current NMA: a detailed process that has been reported elsewhere [17]. The final NMA contained data from 16 RCTs on the relative effectiveness of high compression systems for the treatment of venous leg ulcers. Data for two of the 16 included RCTs (VenUS I and VenUS IV, hereby denominated studies 1 and 2) had full IPD data available (841 participants) which included time to healing or censoring for each participant, together with other individual-level characteristics such as treatment centre, ulcer duration and size and also patient mobility. For the remaining RCTs (1105 participants), aggregate data on the number of healed ulcers were extracted from the source review alongside information regarding treatment type, number of participants allocated to each treatment group, mean duration of follow-up (if this was not stated, trial duration was used), mean ulcer duration and size.

The 16 included RCTs described nine unique high compression treatments: the five standard treatments (4LB, SSB, ZINC, HH and the 2LB) and four *ad hoc* systems [17]. The *ad hoc* group consisted of treatments deemed irrelevant to current clinical practice, and are not reported further (results can be provided upon request). These studies were, however, included in the NMA as their data was potentially relevant, for example, in describing determinants of healing.

**Figure 1:**
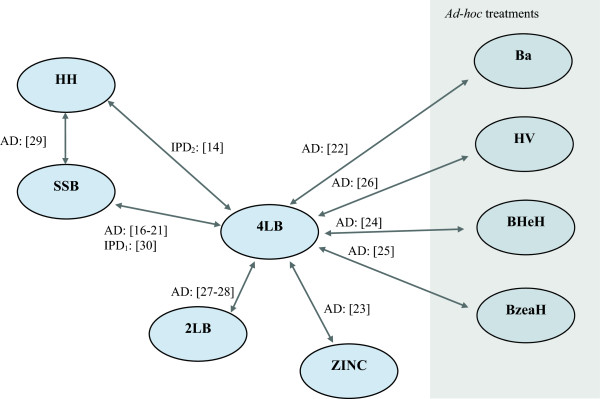
**Network of RCTs.** In the network, a unique treatment category is indicated by a circle. Arrows between circles indicate that these treatments had been compared in a trial (trials are identified using ‘[]’, numbered as in column ‘ID’ in Table 1. (4LB, SSB, HH, Ba, Zinc Paste, BHeH, BzeaH, HV and 2LB as described in Additional file 1).

Table 1 describes the data available and Figure 1 presents the treatment network formed by the evidence [17-32]. The most populated comparison was the 4LB vs. SSB, informed by seven RCTs: six with healing data available as AD [18-22,31] and one as IPD [32]. The link between the 2LB and 4LB was informed by two RCTs and each of the remaining six comparisons in the NMA were informed by AD extracted from one RCT for each comparison (Table 1).

**Table 1:** Analytic dataset

**ID**	**Study**	**Treatment**	**Follow up (weeks)**	**Number patients**	**Mean duration (months)**	**Mean size (cm**^**2**^**)**	**Number healed**	**Evidence format available**
16	Duby *et al.* 1993 [18]	4LB	12	25	20.5	11.9	11	AD
		SSB	12	25	26.7	13.1	10	
17	Scriven *et al.* 1998 [19]	4LB	52	32	13	13.3	17.6	AD
		SSB	52	32	21	8.3	18.24	
18	Partsch *et al.* 2001 [20]	4LB	16	53	1.25	1.5	33	AD
		SSB	16	59	1	1.9	43	
19	Ukat *et al.* 2003 [21]	4LB	12	44	--	17.7	13	AD
		SSB	12	45	--	12.2	10	
20	Franks *et al.* 2004 [22]	4LB	24	74	2	5	59	AD
		SSB	24	82	2	3.5	62	
21	Junger *et al.* 2004b [23]	SSB	12	60	5.57	5.95	19	AD
		HH	12	61	4.14	5.62	29	
22	Kralj *et al.* 1996 [24]	4LB	24	20	7.9	18.6	7	AD
		*Ad hoc*: Ba	24	20	6.9	17.2	8	
23	Polignano *et al.* 2004b [25]	4LB	24	39	--	10.1	29	AD
		ZINC	24	29	--	9.3	19	
24	Wilkinson *et al.* 1997 [26]	4LB	12	17	--	11.2	8	AD
		*Ad hoc*: BHeH	12	18	--	8.6	8	
25	Colgan *et al.* 1995 [27]	4LB	12	10	9.3	27.5	6	AD
		*Ad hoc*: BzeaH	12	10	66.5	48.5	7	
26	Blecken *et al.* 2005 [28]	4LB	12	12	--	50.08	4	AD
		*Ad hoc*: HV	12	12	--	48.98	4	
27	Moffatt *et al.* 2008 [29]	4LB	4	42	48.8	5.7	3	AD
		2LB	4	39	46.6	11.8	6	
28	Szewczyk *et al.* 2010 [30]	4LB	12	15	--	6	9	AD
		2LB	12	16	--	5.3	10	
29	Wong *et al.* 2012 [31]	4LB	24	107	--	--	72	AD
		SSB	24	107	--	--	77	
30	Iglesias *et al.* 2004 [32]	4LB	52	195	3	3.81	107	IPD
		SSB	52	192	3	3.82	86	
14	Ashby *et al.* 2013 [17]	4LB	52	224	12.29	9.30	157	IPD
		HH	52	230	10.82	9.41	163	

AD – aggregate-level data; IPD – individual patient data; 4LB, SSB, HH, Zinc paste,2LB and the *ad hoc* systems Ba, BHeH, BzeaH, HV as described in Additional file 1.

## Methods

We first describe the modelling framework for our main analysis, *model A*. This model brings together IPD and AD into the same synthesis model, with both types of evidence contributing to the estimation of all key models parameters (i.e. absolute and relative effectiveness estimates). We then detail the evaluation of alternative assumptions applied to *model A*, thus highlighting and challenging specific assumptions of the modelling framework proposed. All synthesis was conducted in a Bayesian framework.

Suppose there are two sets of studies, one set for which IPD are available and one set for which AD are available. Consider the set of studies to be T = {1,…, number of IPD studies (NS_IPD_),…, NS_IPD_ + number of AD studies (NS_AD_)} where the total number of studies is *J*; consider the set of treatments to be {1,2,3,…}, where the total number of treatments is *K*, and treatment 1 the reference.

### Statistical model for the data

We describe *model A* in two interrelated parts: *part I* describes the modelling of the IPD and *part II* the modelling of the AD.

(A1) $$\begin{array}{l} \text{Model}\;A,\text{part}\;I-\;\text{modelling}\;\text{the}\;\text{IPD}\;\text{studies},\\ \;\text{controlling}\;\text{for}\;\text{baseline}\;\text{covariates}\\ \begin{array}{c} t\_\text{ijk}∼\text{Weilbull};\left(s,\lambda_{\text{ijk}}\right)I\left(t_{\text{ijk}}^{c}\right)\\ \text{log}\left(\lambda_{\text{ijk}}\right)=\left\{\begin{array}{cc} \mu_{j}^{\text{IPD}}+\gamma_{j}^{c}+\beta_{0j}x_{\text{ijk}} & \text{if}\;k=\;b\\ \mu_{j}^{\text{IPD}}+d_{\text{bk}}+\gamma_{j}^{c}+\beta_{0}x_{\text{ijk}} & \text{if}\;k>\;b \end{array}\right)\\ x\_\text{ijk}∼N\left(m,p\right)\gamma_{j}^{c}∼N\left(0,\pi \right) \end{array} \end{array}$$

Time to ulcer healing (*t*_*ijk*_) of the *i*^*th*^ participant in the *j*^*th*^ study (where *j =1,…, NS*_*IPD*_) and in the *k*^*th*^ treatment arm was assumed to be Weibull distributed [33] with shape^a.^ parameter, *s*, and scale, *λ*_*ijk*_. For some participants, time to event was not observed, and these observations were censored at the time the participant last had trial data recorded, $t_{\text{ijk}}^{c}$. The linear predictor log(*λ*_*ijk*_), was modelled as a function of the log-hazard of an event for the baseline treatment *b*, $\mu_{j}^{\text{IPD}}$, of the log of the treatment effects, *d*_*bk*_ and of a study-specific individual-level regression term, *β*_0*j*_*x*_*ijk*_, where *β*_0*j*_ exemplifies a covariate effect, i.e. the difference in the log hazard ratio per unit increase in the covariate *x*_*ijk*_ a patient-level covariate for the *i*^*th*^ patient in the *j*^*th*^ trial on treatment *k* available in the IPD data sets [17]). The effect of each covariate on the hazard of healing was assumed to be equal in both IPD studies. Due to the possibility of missing covariate information existing for some individuals, *x*_*ijk*_ was represented as a Normally distributed random variable with mean *m* and precision *p*, common across all IPD studies. This is essentially a multiple imputation technique through MCMC^b.^ and assumes that the missingness mechanism was at random^c.^. Additionally, to account for centre variability within each IPD study, $\gamma_{j}^{c}$ was defined for each centre, *c*, in the *j*^*th*^ study, these were combined using a common frailty effect described by a normal distribution with mean zero and precision *τ*.

The treatment effects, *d*_*bk*_, were log-hazard ratios for treatment *k* relative to the study-specific baseline treatment *b*, partitioned further as *d*_1*k*_ - *d*_1*b*_. This equation defines the set of functional parameters, that is the log effects of each treatment in relation to the reference treatment 1. Note that *d*_11_ = 0, where treatment 1 was 4LB, arbitrarily chosen as reference treatment.

Vague prior distributions were specified for $\left[\mu_{b}^{\text{IPD}}\left(∼N\left(0,10^{6}\right)\right)\right]$ for the regression coefficient *β*_0*j*_ (∼*N*(0, 10^6^)) for *m*(∼*N*(0, 10^6^)) and *p*(∼*Gamma*(0.01, 0.01)) for the shared centre effect *τ*(∼*Gamma*(0.01, 0.01)), for *s*(∼*Gamma*(0.01, 0.01)) and for *d*_1*k*_ ∼ *N*(0, 10^6^).

(A2) $$\begin{array}{l} \text{Model}\;A,\;\text{part}\;\text{II}\;-\;\text{modelling}\;\text{the}\;\text{AD}\;\text{studies}\\ \;\begin{array}{c} r\_\text{jk}∼\text{Bin}\left(p_{\text{jk}},n_{\text{jk}}\right)\\ p_{\text{jk}}\;=\;1-\text{exp}\left(-\lambda_{\text{jk}}^{\text{AD}}.{\left(t_{\text{jk}}^{\text{AD}}\right)}^{s}\right)\\ \text{log}\left(\lambda_{\text{jk}}^{\text{AD}}\right)=\left\{\begin{array}{cc} \mu_{j}^{\text{AD}} & \text{if}\;k\;=\;b\\ \mu_{j}^{\text{AD}}+d_{\text{bk}} & \mathrm{if}\;k\;>\;b \end{array}\right) \end{array} \end{array}$$

Within the AD studies, the observed number of participants with a healed ulcer within each study, *r*_*jk*_, out of the total number of individuals in the *j*^*th*^ trial [(where *j = NS*_*IPD*_*+1,…, (NS*_*IPD*_*+ NS*_*AD*_)] and *k*^*th*^ treatment (intention to treat), *n*_*jk*_, was assumed to be Binomially distributed. The underlying probabilities of an event for each arm and in each trial were represented by *p*_*jk*_. In turn, *p*_*jk*_ was expressed as a function of the scale parameter, $\lambda_{\text{jk}}^{\text{AD}}$, of follow-up time, $t_{\text{jk}}^{\text{AD}}$, and the shape parameter, *s,* of the Weibull distribution. The linear predictor, $\log\left(\lambda_{\text{jk}}^{\text{AD}}\right)$ was a function of the baseline log-hazard of an event for treatment *b* in study *j*, $\mu_{\text{jb}}^{\text{AD}}$, and by the log-hazard ratio for treatment *k* and baseline treatment *b*, *d*_*bk*_(=*d*_1*k*_ - *d*_1*b*_) Note that there are parameters common to both model parts (equations A1 and A2), namely the log-hazard ratios and the shape parameter of the time to healing distribution. Vague prior distributions were specified for $\mu_{\text{jb}}^{\text{AD}}\left(∼N\left(0,10^{6}\right)\right)$.

### Alternative modelling assumptions

Underlying *model A* are a set of assumptions that are detailed below. Such assumptions have been relaxed in *models B to D*.

#### *Exploring between-study variation*

*Model A* assumed that each included RCT aimed to measure a common treatment effect (fixed-effect); however, it is likely that there was between-study variation. *Model B* included a random effect to characterise between-study heterogeneity, where *d*_*bk*_ was replaced by a study specific *δ*_*jbk*_, that is then described by a Normal distribution with mean *d*_*bk*_ and precision, *prec* – this is common to both *parts I* (eq. A1) and *II* (eq. A2).

#### *Time to healing distributions*

*Model A* used the Weibull distribution to describe time to healing. Our choice of survival distribution was limited as distributions such as the Log-Logistic or the Log-Normal do not allow the probability of healing over time to be expressed in a closed form, and hence impede the approach proposed here for the joint synthesis of IPD and AD. Other distributions, such as the Gompertz, were not readily defined within the software used in this work (WinBugs/OpenBugs), specifically under censoring. Nonetheless, the goodness of fit could still be assessed in each IPD data source individually. To do this, we applied parametric regression survival-time models [33] to both IPD data sources [17,32] independently (covariates and frailty effect considered, as in *model A*).

#### *Distributional shape parameter*

*Model A* assumes that the Weibull shape parameter of the hazard of healing was common to both IPD data sources. It is possible that this parameter differed between studies, in which case HRs could be affected. Thus, we implemented two alternative NMA models to ascertain the impact of this assumption on the relative effectiveness estimates: *model C1* used the shape parameter from the first IPD study to describe the AD studies and *model C2* used the shape parameter from the second IPD study to describe these same studies. Because *models C1* and *C2* represent simple modifications of *model A* we do not present these algebraically.

#### *Treatment-covariate associations*

*Model A* uses baseline covariates to adjust for clinical heterogeneity in the IPD. To further explore the impact of covariates on the relative treatment effects (i.e. whether they were effect modifiers), and potentially help explain between-study heterogeneity, we also included interaction terms between alternative treatments and baseline ulcer area and duration— as described by Cooper *et al.* 2009 [34] and Saramago *et al.* 2012 [6]. *Model D* assumed a regression (slope) coefficient for the interaction terms, this effect was common across treatments and thus common to *parts I* (eq. D1) and *II* (eq. D2). This assumption was data driven, as this was the only option we were able to implement with the data available (i.e. compared to assuming ‘exchangeability’ or ‘independence’). Note that interaction estimates obtained were influenced by the full evidence base for which study mean covariate(s) values were available, including trials considering *ad hoc* treatments.

Model D, part I- modelling the IPD studies, considering common treatment-by-covariate interactions

(D1) ModelD,partI-modellingtheIPDstudies,consideringcommontreatment-by-covariateinteractionst_ijk∼Weibull;s,λijkItijkclogλijk=μbIPD+γjc+β0jxijkifk=bμbIPD+γjc+β0jxijk+dbk+βxijkifk>bx_ijk∼Nm,pγjc∼N0,τ

Time to ulcer healing was modelled in the same way as in *model A* (equation A1). A treatment-by-covariate interaction regression term, *βx*_*ijk*_, was defined, where *β* is the association effect, assumed common across studies and the same regardless of treatment (excluding control). We included no interaction term for each comparison of *k* versus *b* when *b ≠ 1* because the common regression coefficient cancels out; but, as *β*_11_ = 0, we included an interaction term for each comparison of *k* versus treatment *1*. For the remainder of the parameters of interest, vague prior distributions were assigned as in *model A*.

(D2) ModelD,partII-modellingtheADstudies,consideringcommontreatment-by-covariateinteractionsr_jk∼Binpjk,njkpjk=1-exp-λjkAD.tjkADslogλjkAD=μjbADifk=bμjbAD+dbk+βx¯jifk>b

In part two of *model D*, ${\overset{¯}{x}}_{j}$ represented the mean covariate value for the *j*^*th*^ trial ((where *j = NS*_*IPD*_*+1,…, (NS*_*IPD*_*+ NS*_*AD*_)). Both the IPD and AD contributed to the estimation of this interaction term (i.e. same *β* term in equations D1 and D2). All other components of the model were as described for *model A*.

### Model selection and implementation

The NMA analyses were undertaken in the WinBUGs software [35]. In all models the MCMC sampler was run for 10 000 iterations and these were discarded as ‘burn-in’. Models were run for a further 5000 iterations, on which inferences were based. Chain convergence was assessed by running the model for two disparate sets of initials values, and by then checking that the Brooks-Gelman-Rubin diagnostic converges to 1.0. The WinBUGS code is included for reference in Additional file 2. Within the NMA, goodness of fit was assessed using the deviance information criterion (DIC) [36]. Results were presented using hazard ratio estimates (and associated credibility intervals, CrIs) and also using the probability of each compression system being the ‘best’ treatment in terms of being the most clinically effective [37].

The statistical software STATA [38] was used to fit alternative time to event distributions to the IPD datasets individually. Goodness of fit was assessed with the Akaike Information Criteria (AIC) statistic [39].

## Results

**Figure 2:**
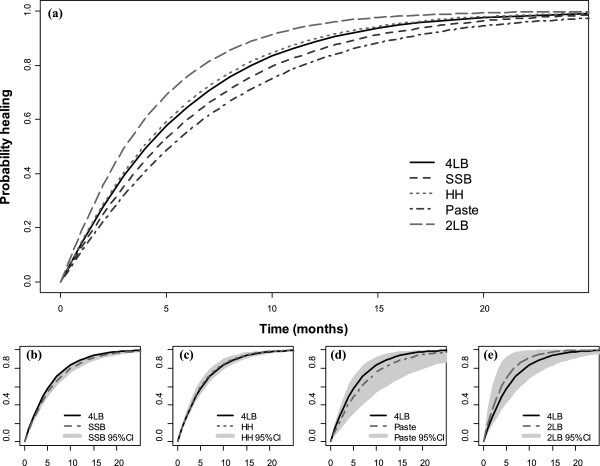
**Graphical representation of model A results reflecting uncertainty over relative treatment effects in the probability of healing over time for the five main high compression ulcer treatments.** The main figure **(a)** shows the expected probabilities of healing (point estimates) across time (25 months); figures **(b)**, **(c)**, **(d)** and **(e)** compare the expected values for four layer bandage with the healing probability (point estimates and uncertainty) of each of the other four high compression treatments. Estimates reflect the average participant in the trial data from VenUS IV (IPD study 2) (mean ulcer area at baseline of 9.4cm2 and ulcer duration at baseline of 11.5 months). (4LB, SSB, HH, Zinc Paste and 2LB as described in Additional file 1).

Table 2 shows parameter estimates obtained for *model A* (first column) and alternative models relaxing its assumptions (*models B* to *D*, second to fifth columns). *Model A* considers the baseline information for the following covariates: the log of the ulcer area (in cm^2^) and duration (in months) - both centred to its sample mean; and dummies generated from a categorical variable on participants’ mobility with three categories (‘immobile’, ‘walks with difficulty’ and ‘walks freely’, the latter being the reference category). The results for *model A* highlight that the modelling framework proposed is feasible. A graphical representation of *model A* results is shown in Figure 2. This plots the probability of healing over time for the five main high compression ulcer treatments, but considers only the uncertainty over relative treatment effects. The results of testing the assumptions are described next.

**Table 2:** Parameter estimates from the alternative MTC synthesis models

		***Model A***			***Model B***			***Model C1***			***Model C2***			***Model D***		
**Hazard ratios**		**HR**	**Median (95% CrI)**	**P**	**HR**	**Median (95% CrI)**	**P**	**HR**	**Median (95% CrI)**	**P**	**HR**	**Median (95% CrI)**	**P**	**HR**	**Median (95% CrI)**	**P**
**Treatment effects**	***4LB***	—	—	5.5	—	—	1.4	—	—	6.2	—	—	5.7	—	—	4.3
	***SSB***	0.88	(0.76, 1.03)	0.4	0.96	(0.77, 1.22)	0.6	0.89	(0.77, 1.04)	0.6	0.89	(0.77, 1.04)	0.6	0.84	(0.70, 0.99)	0.2
	***HH***	1.05	(0.85, 1.29)	16.1	1.63	(0.76, 3.53)	59.2	1.03	(0.83, 1.27)	14.9	1.03	(0.84, 1.27)	15.0	1.03	(0.84, 1.28)	11.1
	***ZINC***	0.77	(0.41, 1.42)	6.2	0.78	(0.37, 1.62)	2.8	0.78	(0.41, 1.44)	6.5	0.78	(0.41, 1.43)	6.7	0.75	(0.03, 29.49)	17.5
	***2LB***	1.40	(0.65, 3.05)	71.9	1.39	(0.62, 3.30)	36.0	1.38	(0.66, 3.05)	71.8	1.38	(0.63, 3.04)	72.0	1.59	(0.61, 5.34)	67.0
**Baseline characteristics**	***Log area***	0.71	(0.66, 0.76)	—	0.71	(0.66, 0.76)	—	0.70	(0.65, 0.75)	—	0.70	(0.65, 0.75)	—	0.71	(0.65, 0.76)	—
	***Log duration***	0.92	(0.90, 0.94)	—	0.92	(0.90, 0.94)	—	0.92	(0.91, 0.94)	—	0.93	(0.91, 0.94)	—	0.92	(0.90, 0.94)	—
	***Difficulty in walking***	0.71	(0.60, 0.85)	—	0.73	(0.60, 0.86)	—	0.72	(0.60, 0.85)	—	0.72	(0.60, 0.85)	—	0.71	(0.60, 0.80)	—
	***Immobile***	0.67	(0.23, 1.52)	—	0.66	(0.23, 1.51)	—	0.72	(0.24, 1.65)	—	0.72	(0.25, 1.67)	—	0.68	(0.24, 1.59)	—
**Interactions**	***Log area***	—	—	—	—	—	—	—	—	—	—	—	—	1.00	(0.97, 1.10)	—
	***Log duration***	—	—	—	—	—	—	—	—	—	—	—	—	1.00	(0.99, 1.00)	—
	***Btw-centre SD***	0.04	(0.01, 0.13)	—	0.05	(0.01, 0.13)	—	0.05	(0.01, 0.15)	—	0.05	(0.01, 0.15)	—			—
	***Btw-study SD***	—	—	—	0.13	(0.01, 0.51)	—	—	—	—	—	—	—	—	—	—
	***λ***	1.07	(1.01, 1.13)	—	1.07	(1.01, 1.14)	*λ*_1_**	0.93	(0.86, 1.01)	*λ*_*1*_	0.93	(0.86, 1.01)	—	1.07	(1.01, 1.14)	—
							*λ*_*2*_	1.27	(1.17, 1.38)	*λ*_2_**	1.27	(1.17, 1.38)				
	***DIC***	5396.2			5396.2			5371.2			5371.5			5377.4		

*Model A* – Fixed-effects NMA, Weibull model of IPD+AD; *model B* – Random-effects NMA, Weibull model of IPD+AD; *model C1* - Fixed-effects NMA, Weibull model of IPD+AD, shape parameter derived from IPD study 1 only; *model C2* - Fixed-effects NMA, Weibull model of IPD+AD, shape parameter derived from IPD study 2 only; *model D* - Fixed-effects NMA, Weibull model of IPD+AD, considering 2 treatment-effect modifiers.

**Shape parameter used in the synthesis model section for summary data.

4LB = four layer bandage; SSB = Short stretch bandage; HH = two layer hosiery; 2LB = two layer bandage.

HR = hazard ratios; CrI = credibility interval; SD = standard deviation; P = probability of being the best treatment choice in terms of healing (%); DIC – deviance information criteria.

### Exploring between-study variation

Despite estimates of HRs from the random effect model (*model B*) being associated with wider CrIs than those from *model A* (as expected), point estimates were found to be fairly similar except for the comparison between HH *vs.* 4LB: HH is estimated to be more effective in *model B* (HR 1.63, 95% CrI 0.76-3.53) compared to *model A* (HR: 1.05, 95% CrI 0.85 to 1.29), although the CrI of the former includes the later. The treatment with the greatest estimated probability of healing was HH in *model B* (59%), rather than 2LB (72%) as in *model A*. Differences may be explained by any existing variation between studies of SSB *vs.* 4LB indirectly impacting on the evidence loop 4LB *vs.* SSB *vs.* HH. Baseline covariate effect estimates remained similar. However, note that the gain in quality fitting of the random-effects model compared to the fixed-effects is null (DIC: 5396.21 and 5396.22, respectively). Previous published work assessing evidence on the SSB *vs.* 4LB comparison [40] similarly found no evidence of between-study heterogeneity.

### Time to healing distributions

The Weibull was used to describe time to healing in *model A*. Whilst we were limited in the use of other distributions, goodness of fit was explored by applying alternative time to event distributions to the IPD studies individually. Table 3 shows results of such analysis (AIC statistic) and Additional file 3 graphically presents the fitted curves and the Kaplan Meier counterpart for control arms. The best fitting distributions for both studies were the Log-Logistic and Log-Normal. Of the remaining, the Weibull and Gompertz distributions provided better fit than the Exponential; this was expected given the flexibility of these distributions in assuming increasing, decreasing or constant hazards over time. The Weibull was best in IPD study 2 and the Gompertz best in IPD study 1.

**Table 3:** Goodness of fit (AIC statistics) of alternative time to ulcer healing models for IPD studies 1 and 2

***Time to event model***	**Akaike Information Criteria (AIC)**	
	**IPD study 1 (30)**	**IPD study 2 (14)**
***Weibull PH***	1102.1	1021.0
***Gompertz PH***	1072.5	1065.5
***Exponential PH***	1102.7	1068.4
***Log-Logistic AFT***	1026.1	971.8
***Log-Normal AFT***	1032.2	961.5

PH = proportional hazards model; AFT = accelerated failure time model

### Distributional Weibull shape parameter of healing hazard

The Weibull shape parameters estimated within *models C1* and *C2* indicate that in IPD study 1 [32] the hazard of healing was expected to decrease over time (*s*_*1*_ = 0.93, 95% CrI 0.86-1.01), while in IPD study 2 it is expected to increase (*s*_*2*_ = 1.27, 95% CrI 1.17-1.38). Note that there is no overlap in the CrIs. However, results show that relative effectiveness estimates are robust to the range of assumptions tested: the estimated HRs did not differ between *models C1* and *C2*, and did not substantially differ from *model A*.

### Treatment-covariate associations

*Model D* tested the inclusion of interaction terms. Two covariates on ulceration area (in cm^2^) and duration (in months) were considered and their association with treatment evaluated. Results (column 5 of Table 2) show that the covariates included did not appear to be treatment effect modifiers in this case study. However, estimating these two additional regression terms increased uncertainty in relative treatment effects estimates, specifically for ZINC and 2LB.

## Discussion

This paper introduces a novel NMA modelling approach that allows IPD on time to event (with censoring) and AD on event count (for a given follow-up time) to be synthesised jointly, by assuming an underlying, common, distribution of time to healing. Available IPD is used directly to inform this distribution (likelihood). Studies reporting the number of participants healed (AD) are used to inform a probability parameter, and a Binomial likelihood was defined for this subset of the evidence-set. The probability of healing was then related (algebraically) to the common distribution of time to healing, by taking the duration of follow-up in each AD study into account. This modelling framework extends approaches in the literature [5,14,15] and is also a natural extension of previously published methodologies of synthesising IPD and AD jointly [6-8,16]. This work was motivated by a real data example looking at the effectiveness of high compression treatments on the healing of venous leg ulcers.

We found that the key strength of the use of IPD in this context (additional to the known advantages described in the introduction) was the flexibility in modelling these data allowed. For example, had all evidence been available as AD, the modelling process would have been limited to the specification of uniparametric distributions for time to healing i.e. the Exponential, with constant healing hazard over time [5,14,15]. In our motivating example, the Exponential distribution was shown to be less adequate than other distributions in describing the time to event data in the studies for which IPD were available. The availability of IPD allowed a more complex distribution for time to event outcomes to be implemented, in this case the Weibull. This may be of particular importance when absolute effectiveness estimates, and not just relative effect estimates, are of interest – and especially where results may need to be extrapolated beyond the follow-up time horizon.

We note that even with the flexibility offered by the use of IPD we were, in practice, limited to using the Weibull distribution. Semi-parametric methods are also unmanageable as the evidence in aggregate form consists of a single data point and thus does not allow defining a non-parametric distribution of hazard of healing. Given this limitation, the synthesis of time to event data will still often require the use of potentially suboptimal distributional assumptions, in which case estimates obtained may be biased. We suggest further research could focuses on using numerical analysis techniques within NMA, to try and resolve this issue.

This work was also relevant in again highlighting the importance of including IPD when wanting to consider patient characteristics in the model. In the presence of heterogeneity, incorporating information on either *a)* patients’ baseline characteristics or *b)* control for treatment-effect modifiers within the synthesis model has been shown to improve estimates and, by doing so, possibly resolve evidence inconsistencies [6,34]. The first relates to potential heterogeneity in the baseline hazard, which cannot be explicitly explored with AD alone (this is important when analyses aim to explore determinants of baseline hazard, for example). In this study we assumed a common effect of baseline covariates on the hazard of healing across IPD studies (as commonly assumed in IPD meta-analysis). The second relates to treatment-covariate interactions, which are generally acknowledged to be best estimated using IPD, as ecological bias can be avoided [9]. For the proportion of evidence only available as AD, the model here implemented considered study level mean covariate values. Nonetheless, not all studies provided information for these, and imputation was undertaken (imputation is naturally done through the MCMC, and assumes values are ‘missing at random’).

In our work we assumed the shape parameter of the Weibull time to event distribution to be common across studies. However, testing proved this assumption was not valid, thus highlighting the importance of evaluating any assumptions of similarity imposed across studies. Despite relative effectiveness estimates being mainly unaffected, such potential heterogeneity between studies should be explored and accounted for in analyses. Such assumptions of commonality also mean that information may be shared throughout the network, in which case evidence on treatments other than those on our decision set (the five treatments of interest for which results were reported). This is the case of *model D* that makes use of all evidence (including *ad hoc* treatments) to estimate treatment-covariate interaction, which may indirectly affect the relative effectiveness estimates of interest.

In the network of evidence there was one closed loop where both direct and indirect data informed relative treatment effect estimates. The existence of inconsistency was explored elsewhere [17], showing no evidence of statistically significant discrepancies between the direct and the indirect data. We note, however, that given the fairly high uncertainty in the evidence base, only large differences in direct and indirect data within the loop would have returned a statistically significant result.

The evidence included as AD in our case study was limited to the primary outcome data on proportion of patients healed at the end of follow-up. However, we could have extended the modelling framework to use any other summary data reported, such as number of patients healed at different time points or Kaplan-Meier (KM) curves. Information conveyed in Kaplan Meier plots can be reconstructed [41], and further research could focus on including this information in our NMA modelling framework to strengthen inferences.

## Conclusions

The use of IPD for a time to event outcome is particularly useful in guiding HTA decision making by allowing flexibility in the specification of more appropriate survival distributions and in dealing with potential existing study heterogeneity [42]. Increasingly, data sharing is promoted in research [43,44] and this example highlights how use of IPD allows the development of more informative and flexible models that are better able to summarise existing evidence. However, it is important to acknowledge that accessing and analysing IPD can be time consuming and may cause delay [4]. The process needs to be well planned and implemented. In our case both sources of IPD were easily accessed and that directly facilitated the conduct of these analyses and the associated methodological work presented here.

## Endnotes

^a.^The shape parameter of the Weibull distribution, *s*, can be interpreted directly as follows: i) if 0 <*s* < 1, hazard rate decreases over time; ii) if *s* = 1, hazard rate is constant over time (hazard exponentially distributed); and if *s* > 1, it indicates that the hazard increases with time.

^b.^When imputing missing information, MCMC generates independent draws of the missing data from its predictive distribution. Multiple imputation through MCMC techniques is attractive for exploratory or multi-purpose analyses involving a large number of estimands.

^c.^The missing-at-random assumption (sometimes called the ignorability assumption) considers that the probability that an observation is missing may depend on the observed values but not the missing values, as sufficient data has already been collected.

## Abbreviations

2LB: Two layer bandage; 4LB: Four layer bandage; AIC: Akaike information criteria; AD: Aggregate data; DIC: Deviance information criteria; HH: Two layer hosiery; HTA: Health technology assessment; IPD: Individual patient-level data; MCMC: Markov chain Monte Carlo; NMA: Network meta-analysis; RCT: Randomised controlled trial; SSB: Short stretch bandage; ZINC: Zinc paste bandage.

## Competing interests

The authors declare that they have no competing interests.

## Authors’ contributions

PS developed and implemented the novel synthesis models, conducted the analysis and prepared the final manuscript. L-HC structured the evidence base and conducted preliminary data analysis using existing published synthesis methodology. MS oversaw and advised on all elements of the work undertaken and prepared the final manuscript. All authors read and approved the final manuscript.

## Pre-publication history

The pre-publication history for this paper can be accessed here:

http://www.biomedcentral.com/1471-2288/14/105/prepub

## Supplementary Material

Additional file 1 Description of main compression systems evaluated. Click here for file

Additional file 2 WinBUGS code. Click here for file

Additional file 3 Observed and fitted survival distributions for IPD studies 1 and 2. Click here for file
