# Supplementary material for: Network meta-analysis of (individual patient) time to event data alongside (aggregate) count data
Source: BMC Med Res Methodol. 2014 Sep 10;14:105. doi: 10.1186/1471-2288-14-105 (PMC4236567; doi:10.1186/1471-2288-14-105)
Supplement: Additional file 1 — Description of main compression systems evaluated. [file 1471-2288-14-105-S1.docx]

## Appendix

**Box A1:** Description of main compression systems evaluated

1. Two layer hosiery, HH (smooth first layer, or understocking, providing light compression over which a second overstocking i.e. UK class II or III depending on the understocking slips on);

2. Four layer bandage, 4LB (an elastic system consisting of an orthopaedic wool layer plus three subsequent bandages);

3. Short stretch bandage, SSB (an inelastic bandage system where one to three rolls of bandage are applied over orthopaedic wool);

4. Zinc paste bandage, ZINC Paste (an inelastic system consisting of a paste bandage often with a support bandage on top);

5. Two layer bandage system, 2LB (bottom layer with cohesive compression bandage - sub-compression wadding layer and cohesive bandage).

### WiNBUGS code

This code relates to *model A* described above and is here described in a generic form for it to be easy for the user to modify and adapt to specific applications. Five datasets are required to fit the complete model: two containing constants for AD and IPD, two for both studies at IPD level and one for the AD evidence. All data should be loaded before the model is compiled. Because of size and agreements of use, the original data sets are not included in their entirety, but a couple of lines of data are supplied for each study/data combination for illustration purposes.

model {

*### Part 1: Model for IPD 1 and IPD 2###*

for(i in 1:n.subjects1) {

*#Weibull likelihood for IPD 1*

t.obs1[i] ~ dweib(shape,zu1[i])I(t.cen1[i],)

*#Model for IPD 1*

log(zu1[i]) <- mu1 + betac1[centre1[i]] + d[treat1[i]] - d[baseline1[i]] +

beta0_cov * cov1[i]

cov1[i] ~dnorm(m1,p1)

}

*#Vague priors for IPD 1*

mu1~dnorm(0,1.0E-6)

m1~dnorm(0,1.0E-6)

p1~dgamma(0.01, 1.0E-3)

for (i in 1:C1) { betac1[i] ~ dnorm(0.0,taua)

}

for(k in 1:n.subjects2) {

*#Weibull likelihood for IPD 2*

t.obs2[k] ~ dweib(shape,zu2[k])I(t.cen2[k],)

*#Model for IPD 2*

log(zu2[k]) <- mu2 + betac2[centre2[k]] + d[treat2[k]] - d[baseline2[k]] +

beta0_cov * cov2[i]

cov2[i] ~dnorm(m2,p2)

}

*#Vague priors for IPD 2*

mu2 ~ dnorm(0,1.0E-6)

m2~dnorm(0,1.0E-6)

p2~dgamma(0.01, 1.0E-3)

for (i in 1:C2) { betac2[i] ~ dnorm(0.0,taua)

}

*#Vague priors for baseline patient characteristics effects*

beta0_cov ~ dnorm(0,1.0E-6)

*# Part 2: Model for aggregate data #*

for(i in 1:n.agg.arm) {

*#Binomial likelihood for AD*

r[i]~dbin(pa[i],n[i])

*#Model for AD*

pa[i] <- 1 - exp( - zu.a[i] * pow(a.time[i], shape))

log(zu.a[i]) <- mu.a[a.s[i]] + d[a.treat[i]] - d[a.base[i]]

}

*#Vague priors for AD*

for(j in 1:n.agg.trials) {

mu.a[j]~dnorm(0,1.0E-6)

}

*### Model for combining all estimates of treatment effect #*

*#Vague prior for shape parameter*

shape ~ dgamma(0.01, 1.0E-3)

*#Vague priors for shared centre effect*

betac.new ~ dnorm(0.0,taua)

taua ~ dgamma(0.01, 1.0E-3)

*#Vague prior for basic parameters*

d[1]<-0

for (k in 2:treat) {

d[k] ~ dnorm(0,1.0E-6)

}

}

*### Dataset 1: Constants to define for IPD evidence###*

*# Number of participants in IPD 1 #*

list(n.subjects1 = 386,

*# Number of participants in IPD 2 #*

n.subjects2 = 454,

*# Number of treatments being evaluated*

treat = 9,

*# Number of centres in IPD 1*

C1 = 9,

*# Number of centres in IPD 2*

C2 = 35)

*### Dataset 2: Constants to define for AD evidence###*

*# Number of AD studies #*

list(n.agg.trials = 14,

*# Number of AD study arms #*

n.agg.arms = 28)

### Dataset 3: IPD 1 ###

| treat1[] | baseline1[] | t.obs1[] | t.cens1[] | cov1[] | centre1[] |
| --- | --- | --- | --- | --- | --- |
| 1 | 1 | 3.50 | 0 | 1.95 | 3 |
| 1 | 1 | 2.33 | 0 | 1.94 | 9 |
| 2 | 1 | NA | 11.90 | 2.49 | 4 |
| ... | ... | ... | ... | ... | ... |
| ... | ... | ... | ... | ... | ... |

END

# treat1 = treatment arm (coded 1,2), baseline1 = reference treatment code,

# t.obs1 = time to event in months (under censoring), t.cens1 = time of censoring in months,

# cov1 = continuous covariate of interest (R+), centre1 = trial centre code (coded 1-9)

### Dataset 4: IPD 2 ###

| treat2[] | baseline2[] | t.obs2[] | t.cens2[] | cov1[] | centre2[] |
| --- | --- | --- | --- | --- | --- |
| 1 | 1 | NA | 21.28 | 5.15 | 1 |
| 1 | 1 | 1.15 | 0 | 0.94 | 16 |
| 3 | 1 | 8.41 | 0 | 2.31 | 2 |
| ... | ... | ... | ... | ... | ... |
| ... | ... | ... | ... | ... | ... |

END

# treat2 = treatment arm (coded 1,2), baseline2 = reference treatment code,

# t.obs2 = time to event in months (under censoring), t.cens2 = time of censoring in months,

# cov1 = continuous covariate of interest (R+), centre2 = trial centre code (coded 1-35)

### Dataset 5: AD evidence ###

| a.s[] | a.treat [] | r[] | n[] | a.base[] | a.time[] |
| --- | --- | --- | --- | --- | --- |
| 1 | 1 | 11 | 25 | 1 | 12 |
| 1 | 2 | 10 | 25 | 1 | 12 |
| ... | ... | ... | ... | ... | ... |
| ... | ... | ... | ... | ... | ... |

END

# a.s = study number, a.treat = treatment arm code (coded from 1 to number of treatments),

# r = number of events in trial arm, n = number of patients in trial arm,

# a.base = reference treatment code, a.time = follow-up time of trial (in months)

### Initial values, either need specifying or generating for the below scalars and vectors ###

list(d = c(NA,0,0,0,0,0,0,0,0), mu1 = -1, mu2 = -1, mu.a = c(-1,-1,-1,-1,-1, -1,-1,-1,-1,-1, -1,-1,-1,-1),

beta_cov = 0, shape = 1, betac.new = 0, betac1 = c(0,0,0,0,0, 0,0,0,0), betac2 = c(0,0,0,0,0, 0,0,0,0,0, 0,0,0,0,0, 0,0,0,0,0, 0,0,0,0,0, 0,0,0,0,0, 0,0,0,0,0))
