# Supplementary material for: Network meta-analysis of (individual patient) time to event data alongside (aggregate) count data
Source: BMC Med Res Methodol. 2014 Sep 10;14:105. doi: 10.1186/1471-2288-14-105 (PMC4236567; doi:10.1186/1471-2288-14-105)
Supplement: Additional file 2 — WinBUGS code. [file 1471-2288-14-105-S2.docx]

Figure: Observed and fitted survival distributions for IPD studies 1 and 2.

IPD study 1 IPD study 2
